# “We have to keep moving”: perspectives on the challenges and opportunities in providing mental health services for people on the move in Latin America

**DOI:** 10.3389/fpsyt.2026.1737063

**Published:** 2026-02-25

**Authors:** Maria Laura Chacón, Sonia Brown Da’Silva, Gladys Vásquez Infante, Diana Gómez-López, Cindy Lisbeth Morales Sánchez, Hunter M. Keys, Doris Altuzar, Cristina Romero, Mayner Rogríguez, Jorge Martín, Jean Hereu, Carolina López Ortiz, Mario López-Alba, Reinaldo Ortuño Gutiérrez, Altair Saavedra, Lindsay Salem-Bango

**Affiliations:** 1 Médecins Sans Frontières, Central America and Integrated Office, Mexico City, Mexico; 2 Médecins Sans Frontières, Project GT141, Guatemala City, Guatemala; 3 Médecins Sans Frontières, Project MX102, Mexico City, Mexico; 4 Médecins Sans Frontières, Project MX121, Reynosa, Mexico; 5 Médecins Sans Frontières, Project HN121, Danlí, Honduras; 6 Médecins Sans Frontières, Operational Center Geneva Headquarters, Geneva, Switzerland; 7 Médecins Sans Frontières – Spain, Colombia & Panamá Mission, Bogotá, Colombia

**Keywords:** health care, Latin America, mental health, migrant, people on the move, psychiatric services, psychological services, violence

## Abstract

Navigating considerable risk and uncertainty, including high rates of violence and recent tightening of migration policies, People on the Move (PoM) in Latin America face significant mental health challenges and barriers to care. From 2021 to 2025, Médecins Sans Frontières (MSF) has provided psychological and psychiatric services to PoM in Mexico, Guatemala, Honduras, Costa Rica, and Panama, conducting almost 17,000 consultations since 2024 alone. In our experience, patients face a complex clinical landscape characterized by limited patient-provider interaction time, constantly changing health systems, and inconsistent referral and medication availability, among other challenges. The urgent need to meet basic survival and protection needs often delays attention to mental health. The highly diverse patient population, both from the region and beyond, requires ongoing adaptation to different languages, cultures, and precipitating events and circumstances. In response, MSF adapts a holistic care package including single, brief therapy sessions; group psychoeducation sessions; pediatric recreational activities; cultural mediators; travel kits with psychiatric medication; and trainings for local providers through the Mental Health Gap Action Programme. Additionally, holistic care integrates mental health services with general medical care and social services, while telehealth and digital health promotion enable providers to reach PoM beyond in-person consults. Recent migration policy changes and funding cuts threaten to exacerbate both the mental health of PoM and barriers in service delivery. Ongoing innovation and adaption are essential to support mental health of PoM in a context of evolving and often punitive regional migration policies.

## Introduction

The Latin American migration route, spanning from South America to Canada, is one of the longest and most transited human migration corridors in the world (1). While comprehensive estimates are difficult to calculate, in 2023 and 2024, Panamanian immigration authorities reported 520,085 and 302,203 people who crossed the Darien Gap from Colombia (2). In the same fiscal years, the United States (US) Customs and Border Protection (CBP) reported 2.5 million (2023) and 2.1 million (2024) encounters at the US/Mexico border (2). Historically, migration through the region has largely been fueled by political instability, economic crises, social inequalities, and violence, with people around the world using this corridor in search of safety, security, and better opportunities, predominantly in the US (1).^1^.

This migration corridor is notoriously dangerous. Violence not only compels people to leave their home countries but remains a constant risk along the route itself. PoM suffer physical abuse, sexual violence, kidnapping, and extortion from a range of state and non-state actors (1, 5–8). Armed groups patrol the Darien Gap, a particularly precarious stretch of jungle on the border of Colombia and Panama, and organized crime targets PoM along the entire route (6). At Médecins Sans Frontières (MSF) clinics, our teams regularly hear horrific stories of sexual violence, including being forced to witness the sexual assault of family members and friends, or being forced to examine each other’s genitals. Kidnapping for ransom has also been a common occurrence, with people held in deplorable conditions and beaten until their families pay for their release. As one patient shared, “They made me watch them beat other people and they put me in a room where they were raping girls of about 11 and 13 in front of their parents. When I closed my eyes to avoid seeing it, they hit me on my back with a wooden board.” (9) Entire belongings may also be stolen, leaving them without money, travel documents, or medications. Victimization by law enforcement, a common reported perpetrator of violence and abuse, prevent many from seeking help, making them even more vulnerable to further revictimization (5). In addition to surviving this violence, PoM traverse treacherous terrain through remote areas with little to no services. They face food insecurity, family separation, and other stressors like caring for infants and children in this environment (6, 10).

Navigating such astonishingly high risk and uncertainty, PoM in Latin America contend with significant mental health challenges and barriers to accessing care, needs MSF staff have witnessed firsthand. Since 2021, MSF has provided comprehensive psychological and psychiatric care to PoM in Mexico, Guatemala, Honduras, Costa Rica, and Panama, conducting nearly 17,000 mental health consultations between January 2024 and May 2025 (9). In this perspective piece – based on the reflections of MSF staff throughout the region – we share our experiences providing mental health care to PoM along the migration route in Latin America. In particular, we highlight the obstacles in finding time and space for mental health care while in transit, hindrances to continuity of care, challenges in providing culturally competent care to diverse populations, limitations of local infrastructure, and unique needs of pediatric patients. Finally, we share the impacts of recent migration policy changes and international funding cuts on mental health service delivery for PoM. A Spanish translation of this article is provided in **Supplementary File 1**.

## Overview of services^2^

In Latin America, MSF adopts both preventative and curative approaches to our mental health services and operates primarily via mobile clinics. Our preventative approach focuses on two domains of life (1): at the family and community level, to support and strengthen existing coping mechanisms so that people with mental health needs can maintain their psychosocial well-being; and (2) at the individual and small-group level, where more focused care in the form of basic counseling can be provided (11). We organize group-based psychoeducation sessions on topics like stress management, coping mechanisms, and the importance of mental health, as well as pediatric recreational activities. When possible, we approach families (often a PoM’s main support system in transit) in individual consultations and groups. Moreover, we conduct health promotion both in person and via digital platforms like Instagram, WhatsApp, and TikTok to reduce stigma around mental health, inform PoM of available services, and encourage care-seeking behavior.

Our curative services involve individual and group psychotherapy with psychiatric treatment as needed. Diagnoses are made following the Diagnostic and Statistical Manual of Mental Health Disorders, 5^th^ Edition (DSM-5) (12), and treatments are suggested in discussion with the patient and reviewing local protocols and drug availability. In our clinics, we commonly see trauma-related cases, such as post-traumatic stress disorder (PTSD), acute depression, and anxiety. However, we also treat patients with pre-existing psychiatric conditions like schizophrenia, bipolar disorder, and substance use disorder, for whom treatment gaps may have started in their home country and are only magnified en route. For patients in Mexico City who require more intensive, long-term services, we operate the Center for Comprehensive Care (CAI) for survivors of torture and ill treatment.

## “We have to keep moving”: finding time and space for mental health care on the migration route

In our experience, competing acute needs like physical health and safety and other challenges can make it difficult to find time and space for mental health care while in transit. A common refrain among patients is that, “We have to keep moving.” On the route, PoM are often in a state of heightened vigilance and stress, a physiological adaptation to prioritize survival over reflective, integrative processes. The physical hardships and stress of acquiring basic needs such as food, water, shelter, and safety leave little time for processing potentially traumatic experiences and can worsen mental health symptoms. Delaying their movement towards their final destination can be both logistically challenging and dangerous, depleting financial resources and increasing time-at-risk. PoM may also only spend a couple of hours to days in one place, limiting provider-patient interaction time. For instance, PoM crossing into Honduras from Nicaragua are provided a three-day visa, allowing them just enough time to travel to the northern border. Even without formal time restrictions, PoM risk being left behind if they delay their group. Our teams regularly see and hear about guides preventing anyone in their group from stopping to seek care unless they look visibly ill.

These constraints lead many PoM to visit our clinics only for physical health needs that had become unbearable. Recognizing this dynamic, we seek to integrate physical, mental, and social services into a single, comprehensive visit. Each provider can refer the patient to another service (including mental health) for same-day consultations, allowing factors beyond what they originally sought care for to be addressed. Non-mental health staff are also trained in psychological first aid, increasing opportunities to identify patients in need. In addition to facilitating access to services, our provision of interdisciplinary care aims to prevent re-traumatization of patients from having to repeat their experiences to new providers across multiple days and service points.

The struggle to find time and space for mental health care witnessed by our staff is reflected in our consultations (**Table 1**). Clinics serving more mobile populations report lower proportions of mental health consultations and, of those, higher proportions of acute diagnoses, compared to clinics serving less-mobile populations. In our experience, PoM actively seek mental health services when they can.

**Table 1:** Characteristics of consultations by population mobility, 2024.

Location	Mobility^1^	Mental health consultations		Principal diagnosis^3^		
		Percent of all consultations^2^, % (n)	Percent follow-up, % (n)	Acute stress reaction^4^, % (n)	PTSD, % (n)	Depression, % (n)
Honduras/Nicaragua border	Higher	15.2% (2,325)	5.5% (129)	72.3% (1,588)	8,7% (190)	7.6% (166)
Guatemala	Higher	22.5% (3,178)	12.5% (396)	64.6% (1,798)	8.7% (190)	8.3% (230)
US/Mexico border	Medium-Lower	29.5% (3,332)	49.1% (1,636)	26.7% (452)	17.9% (304)	15.7% (266)
Mexico City^5^	Lower	43.4% (1,205)	44.1% (531)	11.0% (74)	17.5% (118)	21.7% (146)

This table presents data from a selection of our clinics in 2024 to highlight the trends witnessed by our providers. 2024 data is presented due to significant contextual changes in 2025 that are discussed later in the paper and presented in **Table 2**.

^1^Higher mobility indicates that PoM typically stay in the area for a few days maximum; lower mobility indicates PoM often stay for several weeks or months.

^2^“All consultations” combine consultations for primary health care (for physical health needs) and for mental health (combining psychological and psychiatric services).

^3^Principal diagnosis as identified during baseline consultation.

^4^Acute stress reaction is a normal, expected response to an extreme stressor, rather than a mental health disorder.

^5^Excludes data from the Center for Comprehensive Care (CAI) for survivors of torture and ill treatment.

## Challenges to continuity of care

Aside from recognizing the need to integrate mental health care into a comprehensive care package, our staff face challenges in ensuring continuity of care for PoM. In each stage or country of the route, PoM must find new health facilities and navigate different health systems. Given the impetus to keep moving, of those who do seek mental health care, sessions are often limited to one short session, clearly insufficient to address complex mental health needs. After the initial consultation, patients can schedule in-person follow-up consultations, although this is usually only feasible for patients staying in one place for more time, like in Mexico City. For example, in 2024, 44.1% of outpatient consultations in Mexico City (excluding the CAI) were follow-ups, compared to only 5.5% on the Honduras/Nicaragua border (**Table 1**). Though we have adapted our strategies to reach more PoM in active transit, such as providing telehealth follow-up consultations, barriers like access to mobile phones, laptops, and/or internet connectivity remain a challenge. We try to link patients with other MSF care points further along the route, providing resources with clinic locations and, if relevant, coordinating follow-up consultations with the other facility. We also coordinate with other NGOs and local health systems to promote an informal cross-border referral system, so that key migration actors in the region know where they can refer PoM in need of mental health services.

Limited continuity of care can have important impacts on patients needing prescribed psychiatric medication. At our clinics, patients requiring psychiatric medication may receive a “travel kit”, which includes a three-month supply of medications, prescriptions in English and Spanish, a referral sheet with contact information, psychoeducation materials, a safety plan with emergency contacts, and a map of psychiatric services along their route.

Beyond the limited physical access to health services and potential for medications to be lost, stolen, or damaged, patients face changing prescription protocols in each new country they enter. For example, a PoM in an informal camp in Mexico approached our health promotion team asking if we carried a specific anti-psychotic medication. After years of bad side effects from other prescriptions, he finally found one that worked for him. However, since arriving in Mexico, he had been unable to find that specific medication in local pharmacies. His supply was running low, and he was rationing doses. While this patient had access to psychiatrists, pharmacies, and other antipsychotic medications, he no longer had access to the specific medication that worked best for him.

## Challenges in providing culturally competent care

The patient population moving along the Latin American migration route is highly diverse; we have cared for people not only from throughout Latin America and the Caribbean but from as far away as Afghanistan, China, India, Syria, and the Democratic Republic of Congo. Addressing mental health requires careful consideration of culture. The Western, biomedical formulation of mind-body dualism is not a universally shared concept; rather, ideas of the self, personhood, mind, heart, and soul or spirit are often interconnected and conceptually diverse across cultures (13, 14). Culture influences perception of mental health and illness (including stigmatization), articulation (such as through idioms of distress), and explanatory models, as well as coping mechanisms (what forms of care make sense). Strategies that reduce stigmatization of mental health among Venezuelans might not work for Guineans or Palestinians, for example. Through different idioms of distress, a patient may not explicitly say that they have anxiety but nonetheless communicate anxiety symptoms. Patients from Haiti, for example, often communicate mental distress through head or heart idioms, such as “my heart is tight” or “my head is heavy”, idioms that are associated with depression and anxiety (15). Language barriers are common. We incorporate cultural mediators who speak French and Haitian Kreyol but are otherwise limited to using a translation app. We provide training in culturally competent care for our clinic staff, however the wide range of cultures and languages remains challenging.

## Insufficient local infrastructure

Limited referral services and inconsistent medication availability within existing national health systems further challenge care. In most countries along the migration corridor, few primary-level staff are trained to provide psychological or psychiatric care and specialized mental health services for referrals are scarce. Additionally, it remains very difficult to refer patients from our clinics to specialized care, such as psychiatric hospitals, which often require accompanying family members or documentation that many PoM lack. Although some gaps are covered by other NGOs, they are more likely to have psychologists than psychiatrists. Psychiatric services that do exist within local health systems are typically few, limited and highly bureaucratic. While MSF trains local providers in psychiatric care through the World Health Organization’s Mental Health Gap Action Programme (mhGAP), larger investments are needed to improve mental health care capacity of local health systems.

## Unique challenges for pediatric patients

Children face unique mental health challenges in this context, requiring creative, age-specific interventions. In addition to facing violence and insecurity, minors must often adopt caretaker roles within their families. Others travel as unaccompanied minors, compounding their vulnerability on the route. To support their needs, our teams lead pediatric recreational activities in shelters and camps to integrate topics like wellness and emotional regulation in more child-friendly formats. The health promotion team in Reynosa and Matamoros also used the popular book *The Color Monster: A Story About Emotions* to teach children and young people about mental health (16). This strategy later was adapted into a *Diary of Emotions*, a journal aimed at adolescents, and even a theatrical play that involved PoM. Similarly, the mental health team in Reynosa created a stuffed-toy dinosaur named *Benito Psico Rex* to sensitize children about our mental health services (17). *Benito* was later transformed into a story book in Spanish and Haitian Kreyol to educate children about the role of psychologists. In addition to directly helping children with emotional regulation and wellness, these and other strategies have opened pathways for both our staff and the children themselves to discuss mental health needs with parents, contributing to case detection and referral.

## Impact of recent migration policy changes and cuts to international aid

Unfortunately, we anticipate multiple threats to mental health care in the future. Increasingly restrictive and punitive immigration policies do not stop people from migrating but rather drive them into more clandestine and dangerous routes or leave them suspended in a “waiting period,” typically in precarious circumstances such as squatter camps (9). Over the years, we have heard many PoM process the violence they endure as a necessary evil to reach their destination – one defined as a safer life with more opportunities. Now, many are stranded in makeshift structures in informal camps, especially in Mexico City, that lack necessities like electricity, toilets, and potable water and plagued by violence, including assaults, sexual violence, abductions (both mass abductions for ransom and child abductions), and forced gang initiations. Fear of deportation keeps many from leaving their shelter or camp, worsening their mental health and isolating them from potential support networks and services.

The abrupt closure of the US’s CBP One system in January 2025 upended the life plans of thousands of families and individuals who had been waiting upwards of a year for a response to their immigration applications. In the time since, our clinics have witnessed significant shifts in the mental health landscape. In the days immediately following the closure, our team on the US/Mexico border provided emergency psychological first aid in the camps to address the profound distress that erupted. One psychologist in Mexico City shared that prior to the border closures, she most commonly treated cases of PTSD from violence experienced on the route; now, while she still treats trauma-related cases, many patients come to her with depression and anxiety related to new uncertainties. Another colleague on the Mexico/Guatemala border compared it to a grieving process, saying, “The symptoms are increasingly intense … Many of the cases require pharmacological treatment, with a more structured, longer therapeutic process.” (9) While the closure of CBP One is one of many recent policy changes, it marked a shift towards more restrictive migration policies regionally that have impacted PoM’s mental health. In the last year, we have seen a higher proportion of follow-up consultations as well as a higher proportion of conditions like depression compared to acute stress reactions (**Table 2**).

**Table 2:** Observed changes in mental health consultations.

Category	Pre-CBP one closure	Post-CBP one closure
	1 Jan 2024–19 Jan 2025	20 Jan 2025–31 Aug 2025
	(n=10,298)^1^	(n=2,281)^1^
Follow-up consultations^2^	26.8% (2,764)	57.8% (1,319)
Principal diagnosis^2, 3^		
Acute stress reaction	52.8% (3,978)	20.6% (198)
Depression	11.1% (834)	21.2% (204)

^1^Number of consultations combine baseline and follow-up consultations for psychological and psychiatric services at clinics on the US/Mexico border and in Mexico City, Guatemala, and Honduras.

^2^% (n).

^3^Principal diagnosis identified during baseline consultation.

Meanwhile, recent cuts to international aid have worsened the already limited access to mental health services. While MSF itself has not been directly impacted due to our funding structure, many actors in the region (including national and international NGOs and local health systems) have had to reduce activities or pause them indefinitely (9). This means that in a time of greater mental health stressors, there are fewer services available to PoM. We are concerned this trend will only continue as organizations and local health systems reprioritize their services given funding gaps.

## Conclusion

Our experience providing mental health services along the migration route in Latin America highlights the grave mental health needs of PoM and the stark challenges they face in accessing appropriate care. While many organizations like MSF try to address some of these needs, we cannot reach every person along the corridor. Recent migration policy changes and funding cuts threaten to exacerbate both the mental health of PoM and the barriers in service delivery.

Despite these challenges, providing mental health care for this population is not impossible. Mobile clinics, telehealth solutions, and travel kits are just some of many promising tools for expanding access to mental health services. Integrating mental healthcare into a single-visit, comprehensive care package is essential.

As we reflect on these experiences, we are reminded that mental health care is a critical need and human right. PoM deserve accessible, culturally competent mental health services to ensure their ability to not just survive, but to thrive and rebuild their lives with dignity.

## Data Availability

The datasets presented in this article are not readily available due to ethical restrictions on internal clinical records. Requests to access the datasets should be directed to msfch-mexico-epidemiomanager@msf.geneva.org.

## Footnotes

1 It is important to note that instability in so-called “sending countries” in Latin America is intrinsically intertwined with historical US foreign and domestic policy in the region (3, 4).

2 Due to recent changes in migration, MSF closed its migration clinics in Honduras (May 2025), Guatemala (June 2025), and the US/Mexico border (September 2025). At the time of publication, we continue to provide care to PoM in Mexico City.
