# Supplementary material for: “We have to keep moving”: perspectives on the challenges and opportunities in providing mental health services for people on the move in Latin America
Source: Front Psychiatry. 2026 Feb 25;17:1737063. doi: 10.3389/fpsyt.2026.1737063 (PMC12975999; doi:10.3389/fpsyt.2026.1737063)
Supplement: Supplementary file 1 [file Supplementaryfile1.pdf]

“Tenemos que seguir moviéndonos”: Perspectivas sobre los desafíos y oportunidades en la prestación de servicios de salud mental para personas en movimiento (PeM) en las clínicas de MSF en América Latina

Maria Laura Chacón[1], Sonia Brown Da’Silva[1], Gladys Vásquez Infante[1], Diana Gómez-López[1], Cindy Lisbeth Morales Sánchez[2], Hunter M. Keys[2], Doris Altuzar[3], Cristina Romero[4], Mayner Rogriguez[5], Jorge Martín[3], Jean Hereu[2], Carolina López Ortiz[4], Mario López-Alba[6], Reinaldo Ortuño Gutiérrez[1], Altair Saavedra[7], Lindsay Salem-Bango[1]

[1] Médecins Sans Frontières, Oficina Integrada de Centroamérica y México, Ciudad de México, México
[2] Médecins Sans Frontières, Project GT141, Ciudad de Guatemala, Guatemala
[3] Médecins Sans Frontières, Project MX102, Ciudad de México, México
[4] Médecins Sans Frontières, Project MX121, Reynosa, México
[5] Médecins Sans Frontières, Project HN121, Danlí, Honduras
[6] Médecins Sans Frontières, Centro Operacional de Ginebra, Ginebra, Suiza
[7] Médecins Sans Frontières – España, Misión de Colombia y Panamá, Bogotá, Colombia

Resumen

Al navegar por un riesgo e incertidumbre considerables, incluyendo altas tasas de violencia y el reciente endurecimiento de las políticas migratorias, las personas en movimiento (PeM) en América Latina enfrentan importantes desafíos de salud mental y barreras para la atención. De 2021 a 2025, Médicos Sin Fronteras (MSF) brindó servicios psicológicos y psiquiátricos a PeM en México, Guatemala, Honduras, Costa Rica y Panamá, realizando casi 17,000 consultas solo desde 2024. En nuestra experiencia, los pacientes enfrentan un panorama clínico complejo caracterizado por un tiempo limitado de interacción paciente-proveedor, sistemas de salud en constante cambio y disponibilidad inconsistente de referencias y medicamentos, entre otros desafíos. La necesidad urgente de satisfacer las necesidades básicas de supervivencia y protección a menudo retrasa la atención a la salud mental. La población de pacientes altamente diversa, tanto de la región como de otros lugares, requiere una adaptación continua a diferentes idiomas, culturas y eventos y circunstancias desencadenantes. En respuesta, MSF adaptó un paquete de atención holística que incluye sesiones de terapia breves individuales; sesiones de psicoeducación grupal; actividades recreativas pediátricas; mediadores culturales; kits de viaje con medicación psiquiátrica; y capacitaciones para proveedores locales a través del Programa de Acción para la Brecha en la Salud Mental. Además, la atención holística integra los servicios de salud mental con la atención médica general y los servicios sociales, mientras que la telemedicina y la promoción digital de la salud permiten a los profesionales llegar a las PeM más allá de las consultas presenciales. Los recientes cambios en las políticas migratorias y los recortes de financiación amenazan con agravar tanto la salud mental

de las PeM como las barreras en la prestación de servicios. La innovación y la adaptación continuas son esenciales para apoyar la salud mental de las PeM en un contexto de políticas migratorias regionales cambiantes y, a menudo, punitivas.

Introducción

La ruta migratoria latinoamericana, que se extiende desde América del Sur hasta Canadá, es uno de los corredores migratorios humanos más largos y transitados del mundo (1). Si bien es difícil realizar estimaciones precisas, en 2023 y 2024 las autoridades migratorias panameñas reportaron 520,085 y 302,203 personas que cruzaron el Tapón del Darién desde Colombia (2). En los mismos años fiscales, la Oficina de Aduanas y Protección Fronteriza (CBP) de los Estados Unidos (EE. UU.) reportó 2,5 millones (2023) y 2,1 millones (2024) de encuentros en la frontera entre EE. UU. y México (2). Históricamente, la migración a través de la región ha sido impulsada en gran medida por la inestabilidad política, las crisis económicas, las desigualdades sociales y la violencia, y personas de todo el mundo utilizan este corredor en busca de seguridad, protección y mejores oportunidades, predominantemente en los EE. UU. (1).[1]

Este corredor migratorio es notoriamente peligroso. La violencia no solo obliga a las personas a abandonar sus países de origen, sino que también constituye un riesgo constante a lo largo de la ruta. Las PeM sufren abusos físicos, violencia sexual, secuestros y extorsión por parte de diversos actores estatales y no estatales (1,5–8). Grupos armados patrullan el Tapón del Darién, un tramo de selva particularmente precario en la frontera con Colombia y Panamá, y el crimen organizado ataca a las PeM a lo largo de toda la ruta (6). En las clínicas de Médicos Sin Fronteras (MSF), nuestros equipos escuchan con frecuencia historias horribles de violencia sexual, como ser obligados a presenciar agresiones sexuales de familiares y amigos, o a examinarse los genitales mutuamente. Los secuestros para pedir rescate también son comunes, con personas retenidas en condiciones deplorables y golpeadas hasta que sus familias pagan por su liberación. Como compartió un paciente: "Me obligaron a ver cómo golpeaban a otras personas y me metieron en una habitación donde violaban a niñas de entre 11 y 13 años delante de sus padres. Cuando cerré los ojos para no verlo, me golpearon en la espalda con una tabla de madera" (9). También pueden robarles todas sus pertenencias, dejándoles sin dinero, documentos de viaje ni medicamentos. La victimización por parte de las fuerzas del orden, un perpetrador frecuente de violencia y abuso, impide que muchas personas busquen ayuda, lo que los hace aún más

---

[1] Es importante señalar que la inestabilidad en los llamados “países emisores” de América Latina está intrínsecamente entrelazada con la política exterior e interna histórica de Estados Unidos en la región. (3, 4)

vulnerables a una mayor revictimización (5). Además de sobrevivir a esta violencia, las PeM atraviesan terrenos peligrosos en zonas remotas con escasos o nulos servicios. Se enfrentan a la inseguridad alimentaria, la separación familiar y otros factores estresantes, como el cuidado de bebés y niños en este entorno (6,10).

Enfrentando un riesgo e incertidumbre tan sorprendentemente altos, las PeM en América Latina se enfrentan además a importantes desafíos de salud mental y barreras para acceder a la atención, necesidades que el personal de MSF ha presenciado de primera mano. Desde 2021, MSF ha brindado atención psicológica y psiquiátrica integral a PeM en México, Guatemala, Honduras, Costa Rica y Panamá, realizando cerca de 17,000 consultas de salud mental entre enero de 2024 y mayo de 2025 (9). En este artículo de perspectiva, basado en las reflexiones del personal de MSF en toda la región, compartimos nuestras experiencias brindando atención de salud mental a las PeM a lo largo de la ruta migratoria en Latinoamérica. En particular, destacamos los obstáculos para encontrar tiempo y espacio para la atención de salud mental durante el tránsito, los obstáculos para la continuidad de la atención, las dificultades para brindar atención culturalmente competente a poblaciones diversas, las limitaciones de la infraestructura local y las necesidades específicas de los pacientes pediátricos. Finalmente, compartimos el impacto de los recientes cambios en las políticas migratorias y los recortes de la financiación internacional en la prestación de servicios de salud mental para las PeM.

Descripción general de los servicios[2]

Adoptamos enfoques preventivos y curativos en nuestros servicios de salud mental y operamos principalmente a través de clínicas móviles. Nuestro enfoque preventivo se centra en dos ámbitos de la vida: (1) a nivel familiar y comunitario, para apoyar y fortalecer los mecanismos de afrontamiento existentes, de modo que las personas con necesidades de salud mental puedan mantener su bienestar psicosocial; y (2) a nivel individual y de grupos pequeños, donde se puede brindar una atención más específica mediante asesoramiento básico (11). Organizamos sesiones grupales de psicoeducación sobre temas como el manejo del estrés, mecanismos de afrontamiento y la importancia de la salud mental, así como actividades recreativas pediátricas. Siempre que fue posible, nos acercamos a las familias (que suelen ser el principal apoyo de las PeM durante el tránsito) en consultas individuales y grupales. Además, realizamos actividades de promoción de la salud tanto presenciales como a través de

[2] Debido a los recientes cambios en la migración, MSF cerró sus clínicas de migración en Honduras (mayo de 2025), Guatemala (junio de 2025) y la frontera entre Estados Unidos y México (septiembre de 2025). Al momento de esta publicación, continuamos brindando atención a personas migrantes en la Ciudad de México.

plataformas digitales como Instagram, WhatsApp y TikTok para reducir el estigma en torno a la salud mental, informar a las PeM sobre los servicios disponibles y fomentar la búsqueda de atención.

Nuestros servicios curativos incluyen psicoterapia individual y grupal, con tratamiento psiquiátrico según sea necesario. Los diagnósticos se realizan siguiendo el Manual Diagnóstico y Estadístico de los Trastornos de Salud Mental, 5.ª edición (DSM-5) (12), los tratamientos se sugieren en consulta con el paciente y tras revisar los protocolos locales y la disponibilidad de medicamentos. En nuestras clínicas, atendemos con frecuencia casos relacionados con traumas, como trastorno de estrés postraumático (TEPT), depresión aguda y ansiedad. Sin embargo, también atendemos a pacientes con afecciones psiquiátricas preexistentes, como esquizofrenia, trastorno bipolar y trastorno por consumo de sustancias, cuyas deficiencias en el tratamiento pueden haber comenzado en su país de origen y se agravan con el tiempo. Para los pacientes en la Ciudad de México que requieren servicios más intensivos y a largo plazo, operamos el Centro de Atención Integral (CAI) para sobrevivientes de tortura y malos tratos.

“Tenemos que seguir avanzando”: Encontrar tiempo y espacio para la atención de la salud mental en la ruta migratoria

En nuestra experiencia, las necesidades agudas concurrentes, como la salud física y la seguridad, y otros desafíos, pueden dificultar encontrar tiempo y espacio para la atención de salud mental durante el tránsito. Un refrán común entre los pacientes es: "Tenemos que seguir avanzando". Durante la ruta, las PeM suelen encontrarse en un estado de mayor vigilancia y estrés, una adaptación fisiológica que prioriza la supervivencia sobre los procesos reflexivos e integradores. Las dificultades físicas y el estrés de satisfacer necesidades básicas como alimento, agua, refugio y seguridad dejan poco tiempo para procesar experiencias potencialmente traumáticas y pueden agravar los síntomas de salud mental. Retrasar su traslado hacia su destino final puede ser tanto logísticamente desafiante como peligroso, agotando los recursos financieros y aumentando el tiempo de riesgo. Las PeM también pueden pasar solo un par de horas o días en un mismo lugar, lo que limita el tiempo de interacción entre el proveedor y el paciente. Por ejemplo, a las PeM que cruzan a Honduras desde Nicaragua se les otorga una visa de tres días, lo que les da el tiempo justo para viajar a la frontera norte. Incluso sin restricciones de tiempo formales, las PeM corren el riesgo de quedarse atrás si retrasan a su grupo. Nuestros equipos ven y escuchan con frecuencia sobre guías que impiden que cualquier persona de su grupo se detenga a buscar atención médica a menos que parezca visiblemente enferma.

Estas limitaciones llevan a muchas PeM a acudir a nuestras clínicas únicamente por necesidades de salud física que se han vuelto insoportables. Reconociendo esta dinámica, buscamos incluir servicios físicos, mentales y sociales en una única visita

integral. Cada profesional puede derivar al paciente a otro servicio (incluido el de salud mental) para consultas el mismo día, lo que permite abordar factores que van más allá de la atención inicial. El personal no especializado en salud mental también está capacitado en primeros auxilios psicológicos, lo que aumenta las oportunidades de identificar a los pacientes que los necesitan. Además de facilitar el acceso a los servicios, nuestra atención interdisciplinaria también busca prevenir la revictimización de los pacientes al tener que repetir sus experiencias con nuevos profesionales en varios días y puntos de servicio.

La dificultad para encontrar tiempo y espacio para la atención de salud mental, observada por nuestro personal, se refleja en nuestras consultas (Tabla 1). Las clínicas que atienden a poblaciones con mayor movilidad reportan una menor proporción de consultas de salud mental y, de estas, una mayor proporción de diagnósticos agudos, en comparación con las clínicas que atienden a poblaciones con menor movilidad. Según nuestra experiencia, las PeM buscan activamente servicios de salud mental cuando pueden.

Tabla 1. Características de las consultas según movilidad poblacional, 2024.

Sitio	Mobilidad[1]	Consultas de salud mental		Diagnóstico principal[3]		
		% de todas las consultas, % (n)	% de seguimiento, % (n)	Reacción de estrés agudo[4], % (n)	TEPT, % (n)	Depresión, % (n)
Frontera de Honduras/ Nicaragua	Alta	15.2% (2,325)	5.5% (129)	72.3% (1,588)	8,7% (190)	7.6% (166)
Guatemala	Alta	22.5% (3,178)	12.5% (396)	64.6% (1,798)	8.7% (190)	8.3% (230)
Frontera de EE. UU. / México	Media - Baja	29.5% (3,332)	49.1% (1,636)	26.7% (452)	17.9% (304)	15.7% (266)
Ciudad de México[5]	Baja	43.4% (1,205)	44.1% (531)	11.0% (74)	17.5% (118)	21.7% (146)

Esta tabla presenta datos de una selección de nuestras clínicas en 2024 para resaltar las tendencias observadas por nuestros proveedores. Los datos de 2024 se presentan debido a cambios contextuales significativos en 2025 que se analizan más adelante en el documento y se presentan en la Tabla 2.

[1] Una mayor movilidad indica que las PeM normalmente permanecen en la zona durante unos pocos días como máximo; una menor movilidad indica que las PeM a menudo permanecen en la zona durante varias semanas o meses.

[2] "Todas las consultas" combina consultas de atención primaria de salud (para necesidades de salud física) y de salud mental (combinando servicios psicológicos y psiquiátricos).

[3] Diagnóstico principal identificado durante la consulta inicial.

[4] La reacción de estrés agudo es una respuesta normal y esperada a un factor estresante extremo, más que un trastorno de salud mental.

[5] Excluye datos del Centro de Atención Integral (CAI) para sobrevivientes de tortura y malos tratos.

Desafíos para la continuidad de la atención

Además de reconocer la necesidad de integrar la atención de salud mental en un paquete integral de atención, nuestro personal enfrenta desafíos para garantizar la continuidad de la atención para las PeM. En cada etapa o país de la ruta, las PeM deben encontrar nuevos centros de salud y navegar por diferentes sistemas de salud. Dado el impulso para seguir moviéndose, las sesiones de quienes buscan atención de salud mental suelen limitarse a una breve sesión, claramente insuficiente para abordar necesidades complejas de salud mental. Después de la consulta inicial, los pacientes pueden programar consultas de seguimiento presenciales, aunque esto generalmente solo es factible para pacientes que permanecen en un lugar por más tiempo, como en la Ciudad de México. Por ejemplo, en 2024, el 44.1% de las consultas ambulatorias en la Ciudad de México (excluyendo el CAI) fueron de seguimiento, en comparación con solo el 5.5% en la frontera entre Honduras y Nicaragua (Tabla 1). Si bien hemos adaptado nuestras estrategias para llegar a más PeM en tránsito activo, como ofrecer consultas de seguimiento por telemedicina, las barreras como el acceso a teléfonos móviles, portátiles o conexión a internet siguen siendo un desafío. Intentamos conectar a los pacientes con otros puntos de atención de MSF más adelante en la ruta, proporcionando recursos con la ubicación de las clínicas y, si corresponde, coordinando las consultas de seguimiento con el otro centro. También nos coordinamos con otras ONG y sistemas de salud locales para promover un sistema informal de derivación transfronteriza, de modo que los actores clave en migración de la región sepan dónde pueden derivar a las PeM que necesitan servicios de salud mental.

La continuidad limitada de la atención puede tener un impacto importante en los pacientes que necesitan medicación psiquiátrica prescrita. En nuestras clínicas, los pacientes que requieren medicación psiquiátrica pueden recibir un kit de viaje que incluye un suministro de medicamentos para tres meses, recetas en inglés y español, una hoja de derivación con información de contacto, materiales psicoeducativos, un plan de seguridad con contactos de emergencia y un mapa de los servicios psiquiátricos a lo largo de su ruta.

Además del acceso físico limitado a los servicios de salud y la posibilidad de pérdida, robo o daño de medicamentos, los pacientes se enfrentan a protocolos de prescripción cambiantes en cada nuevo país al que ingresan. Por ejemplo, un paciente de un campamento informal en México se acercó a nuestro equipo de promoción de la salud para preguntar si teníamos un medicamento antipsicótico específico. Tras años de sufrir efectos secundarios graves con otras recetas, finalmente encontró uno que le funcionaba. Sin embargo, desde su llegada a México, no había podido encontrar ese medicamento específico en las farmacias locales. Sus reservas se estaban agotando y estaba racionando las dosis. Si bien este paciente tenía acceso a psiquiatras, farmacias y otros antipsicóticos, ya no tenía acceso al medicamento específico que mejor le funcionaba.

Desafíos en la prestación de una atención culturalmente competente

La población de pacientes que transita por la ruta migratoria latinoamericana es muy diversa; hemos atendido a personas no solo de toda Latinoamérica y el Caribe, sino también de lugares tan lejanos como Afganistán, China, India, Siria y la República Democrática del Congo. Abordar la salud mental requiere una cuidadosa consideración de la cultura. La formulación biomédica occidental del dualismo mente-cuerpo no es un concepto universalmente compartido; más bien, las ideas sobre el yo, la personalidad, la mente, el corazón y el alma o espíritu suelen estar interconectadas y ser conceptualmente diversas entre culturas (13,14). La cultura influye en la percepción de la salud mental y la enfermedad (incluida la estigmatización), la articulación (por ejemplo, mediante expresiones idiomáticas de angustia) y los modelos explicativos, así como en los mecanismos de afrontamiento (qué formas de atención son adecuadas). Las estrategias que reducen la estigmatización de la salud mental entre los venezolanos podrían no funcionar para los guineanos o palestinos, por ejemplo. A través de diferentes expresiones idiomáticas de angustia, un paciente puede no decir explícitamente que tiene ansiedad, pero aun así comunicar síntomas de ansiedad. Los pacientes de Haití, por ejemplo, a menudo comunican angustia mental mediante expresiones idiomáticas que se relacionan con la mente o el corazón, como "mi corazón está apretado" o "mi cabeza está pesada", expresiones idiomáticas que se asocian con la depresión y la ansiedad (15). Las barreras lingüísticas son comunes. Contamos con mediadores culturales que hablan francés y criollo haitiano, pero por lo general, nos limitamos a usar una aplicación de traducción. Ofrecemos capacitación en atención culturalmente competente al personal de nuestra clínica; sin embargo, la amplia gama de culturas e idiomas sigue siendo un desafío.

Infraestructura local insuficiente

Los servicios de derivación limitados y la disponibilidad inconsistente de medicamentos dentro de los sistemas nacionales de salud existentes dificultan aún más la atención. En la mayoría de los países a lo largo del corredor migratorio, poco personal de nivel primario está capacitado para brindar atención psicológica o psiquiátrica y los servicios especializados de salud mental para derivaciones son escasos. Además, sigue siendo muy difícil derivar pacientes de nuestras clínicas a atención especializada, como hospitales psiquiátricos, que a menudo requieren familiares que les acompañen o documentación de la que muchas PeM carecen. Si bien otras ONG cubren algunas brechas, es más probable que tengan psicólogos que psiquiatras. Los servicios psiquiátricos que existen dentro de los sistemas de salud locales suelen ser pocos, limitados y altamente burocráticos. Si bien MSF capacita a los proveedores locales en atención psiquiátrica a través del Programa de Acción para la

Brecha en Salud Mental (mhGAP, por sus siglas en inglés) de la Organización Mundial de la Salud, se necesitan mayor inversión para mejorar la capacidad de atención de la salud mental de los sistemas de salud locales.

Desafíos únicos para los pacientes pediátricos

En este contexto, las infancias enfrentan desafíos únicos de salud mental, lo que requiere intervenciones creativas y específicas para su edad. Además de enfrentar la violencia y la inseguridad, las infancias y adolescencias a menudo deben asumir roles de cuidadores dentro de sus familias. Otros viajan sin compañía, lo que agrava su vulnerabilidad durante la ruta. Para apoyar sus necesidades, nuestros equipos organizan actividades recreativas pediátricas en albergues y campamentos para integrar temas como el bienestar y la regulación emocional en formatos más adaptados a las infancias. El equipo de promoción de la salud en Reynosa y Matamoros también utilizó el popular libro "*El Monstruo de Colores: Una Historia sobre las Emociones*" para educar a infantes y adolescentes sobre salud mental (16). Esta estrategia se adaptó posteriormente a un *Diario de Emociones*, un diario dirigido a adolescentes, e incluso a una obra de teatro que incluía PeM. De manera similar, el equipo de salud mental de Reynosa creó un dinosaurio de peluche llamado *Benito Psico Rex* para sensibilizar a los niños sobre nuestros servicios de salud mental (17). *Benito* se convirtió posteriormente en un libro de cuentos en español y criollo haitiano para educar a los niños sobre el papel del personal de psicología. Además de brindar asistencia directa a los niños sobre la regulación emocional y el bienestar, ésta y otras estrategias han abierto caminos para ambos, nuestro equipo y las infancias mismas, para discutir sus necesidades de salud mental con sus padres, contribuyendo así a la detección y referencia de casos.

El impacto de los recientes cambios en las políticas migratorias y los recortes a la ayuda internacional

Desafortunadamente, anticipamos múltiples amenazas a la atención de la salud mental en el futuro. Las políticas migratorias cada vez más restrictivas y punitivas no impiden que las personas migren, sino que las obligan a tomar rutas más clandestinas y peligrosas o las dejan en espera, generalmente en circunstancias precarias como campamentos improvisados (9). A lo largo de los años, hemos escuchado a muchas PeM procesar la violencia que sufren como un mal necesario para alcanzar su destino, definido como una vida más segura con más oportunidades. Ahora, muchas se encuentran varadas en estructuras improvisadas en campamentos informales, especialmente en la Ciudad de México, que carecen de servicios básicos como electricidad, baños y agua potable, y están plagadas de violencia, incluyendo

agresiones, violencia sexual, secuestros (tanto masivos para pedir rescate como de menores) e iniciaciones forzadas en pandillas. El miedo a la deportación impide que muchas abandonen su refugio o campamento, lo que empeora su salud mental y las aísla de posibles redes y servicios de apoyo.

El cierre abrupto del sistema CBP One de EE. UU. en enero de 2025 trastocó los planes de vida de miles de familias e individuos que llevaban más de un año esperando una respuesta a sus solicitudes de inmigración. Desde entonces, nuestras clínicas han presenciado cambios significativos en el panorama de la salud mental. En los días posteriores al cierre, nuestro equipo en la frontera entre EE. UU. y México brindó primeros auxilios psicológicos de emergencia en los campamentos para abordar la profunda angustia que surgió. Una psicóloga en la Ciudad de México compartió que, antes del cierre de la frontera, trataba principalmente casos de TEPT por la violencia sufrida en la ruta; ahora, aunque sigue atendiendo casos relacionados con traumas, muchos pacientes acuden a ella con depresión y ansiedad relacionadas con las nuevas incertidumbres. Otro colega en la frontera entre México y Guatemala lo comparó con un proceso de duelo, diciendo: "Los síntomas son cada vez más intensos... Muchos de los casos requieren tratamiento farmacológico, con un proceso terapéutico más estructurado y más largo" (9). Si bien el cierre de CBP One es uno de los muchos cambios recientes en las políticas, marcó un cambio hacia políticas migratorias más restrictivas a nivel regional que han afectado la salud mental de las PeM. En los últimos nueve meses, hemos observado una mayor proporción de consultas de seguimiento, así como una mayor proporción de afecciones como la depresión, en comparación con las reacciones de estrés agudo (Tabla 2).

Tabla 2. Cambios en las consultas de salud mental

Categoría	**Pre- cierre de CBP One** 1 Ene 2024 – 19 Ene 2025 (n=10,298)[1]	**Post-cierre de CBP One** 20 Ene 2025 – 31 Ago 2025 (n=2,281)[1]
**Consultas de seguimiento**[2]	26.8% (2,764)	57.8% (1,319)
**Diagnóstico principal**[2,3]		
Reacción de estrés agudo	52.8% (3,978)	20.6% (198)
Depresión	11.1% (834)	21.2% (204)

[1] El número de consultas combina consultas iniciales y de seguimiento para servicios psicológicos y psiquiátricos en clínicas de la frontera entre Estados Unidos y México y en la Ciudad de México, Guatemala y Honduras.

[2] %(n)

[3] Diagnóstico principal identificado durante la consulta basal.

Mientras tanto, los recientes recortes a la ayuda internacional han agravado el ya limitado acceso a los servicios de salud mental. Si bien MSF no se ha visto directamente afectado debido a nuestra estructura de financiación, muchos actores de la región (incluidas ONG nacionales e internacionales y sistemas de salud locales) han tenido que reducir sus actividades o suspenderlas indefinidamente (9). Esto significa

que, en un momento de mayor estrés para la salud mental, hay menos servicios disponibles para las PeM. Nos preocupa que esta tendencia continúe a medida que las organizaciones y los sistemas de salud locales reordenen sus servicios debido a la falta de financiación.

Conclusión

Nuestra experiencia en la prestación de servicios de salud mental a lo largo de la ruta migratoria en América Latina pone de relieve las graves necesidades de salud mental de las PeM y los graves desafíos que enfrentan para acceder a la atención adecuada. Si bien muchas organizaciones como MSF intentan abordar algunas de estas necesidades, no podemos llegar a todas las personas a lo largo del corredor. Los recientes cambios en las políticas migratorias y los recortes de financiación amenazan con agravar tanto la salud mental de las PeM como las barreras en la prestación de servicios.

A pesar de estos desafíos, brindar atención de salud mental a esta población no es imposible. Las clínicas móviles, las soluciones de telemedicina y los kits de viaje son solo algunas de las muchas herramientas prometedoras para ampliar el acceso a los servicios de salud mental. Integrar la atención de salud mental en un paquete de atención integral en una sola consulta es esencial.

Al reflexionar sobre estas experiencias, recordamos que la atención de la salud mental es una necesidad crítica y un derecho humano. Las PeM merecen servicios de salud mental accesibles y culturalmente competentes para garantizar su capacidad no solo de sobrevivir, sino también de prosperar y reconstruir sus vidas con dignidad.

Agradecimientos

Queremos agradecer a todos los pacientes que han compartido valientemente sus historias y experiencias con nosotros a lo largo de los años. También queremos agradecer a Carolina Echeverri (MSF) por su apoyo durante la etapa de conceptualización.

Referencias

1. International Organization for Migration. IOM World Migration Report 2024 (2024). Disponible en: https://worldmigrationreport.iom.int/what-we-do/worldmigration-
report-2024-chapter-3/latin-america-and-caribbean (Fecha de acceso: Octubre 10, 2025).
2. Aljazeera, Jazeera Al. Panama reports sharp drop in irregular migration through Darien Gap (2025). Disponible en: https://www.aljazeera.com/news/2025/1/3/Panama-reports-sharp-drop-in-irregular-migration-through-darien-gap (Fecha de acceso: 13 de Octubre, 2025).
3. Katie A. A History of U.S. Intervention in Latin America and the Caribbean. Encyclopedia Britannica. (2026). Disponible en: https://www.britannica.com/topic/History-of-US-Intervention-in-Latin-America-and-the-Caribbean (Fecha de acceso: 3 de febrero, 2026).
4. Absher S, Grier R, Grier K. The consequences of CIA-sponsored regime change in Latin America. European Journal of Political Economy. (2023) 80:102452. doi: 10.1016/j.ejpoleco.2023.102452
5. Gómez-López D, Salem-Bango L, Tamariz M, Hussain N, Taylor M, Sorrenti S, et al. Interconnected experiences of violence exploring the nexus of sexual violence, migration, and asylum plans for people on the move (PoM) in Latin America. J Hum Traffick Enslav Confl-Relat Sex Viol. (2025) 6:165–92. doi: 10.7590/266644725X17528218685762
6. Roy D. Crossing the darién gap: migrants risk death on the journey to the U.S (2024). Disponible en: https://www.cfr.org/article/crossing-darien-gap-migrants-riskdeath-journey-us (Fecha de acceso: 8 de Septiembre, 2025).
7. International Organization for Migration. International organization for migration. Geneva, Switzerland: US-Mexico Border World’s Deadliest Migration Land Route (2023). Disponible en: https://www.iom.int/news/us-Mexico-border-worldsdeadliest-migration-land-route (Fecha de acceso: 8 de Septiembre, 2025).
8. Ramage K, Stirling-Cameron E, Ramos NE, Martinez SanRoman I, Bojorquez I, Spata A, et al. When you leave your country, this is what you’re in for”: experiences of structural, legal, and gender-based violence among asylum-seeking women at the Mexico-U.S. Border BMC Public Health. (2023) 23:1699. doi: 10.1186/s12889-023-16538-2
9. Médecins Sans Frontières. Unwelcome - The devastating human impact of migration policy changes in the United States. Mexico and Central America - Mexico: Médecins Sans Frontières (2025). Disponible en: https://reliefweb.int/report/Mexico/unwelcome-devastating-human-impact-migration-policy-changes-united-states-Mexico-and-central-america (Fecha de acceso: 25 de Agosto, 2025).
10. Bojórquez I, Infante C, Villanueva-Borbolla MA, Orjuela-Grimm M. Solidarity through food: Coping with food insecurity among adolescent migrants in transit

through Mexico and Central America. Appetite. (2024) 200:107549. doi: 10.1016/j.appet.2024.107549
11. Inter-Agency Standing Committee (IASC). IASC guidelines on mental health and psychosocial support in emergency settings. Geneva: IASC. (2008). doi: 10.1037/e518422011-002.
12. American Psychiatric Association. Diagnostic and statistical manual of mental disorders. Fifth Edition. Arlington, VA: American Psychiatric Association. (2013). doi: 10.1176/appi.books.9780890425596
13. Hinton D, Hinton S. Panic disorder, somatization, and the new cross-cultural psychiatry: the seven bodies of a medical anthropology of panic. Cult Med Psychiatry. (2002) 26:155–78. doi: 10.1023/A:1016374801153
14. Kirmayer LJ. Cultural variations in the response to psychiatric disorders and emotional distress. Soc Sci Med 1982. (1989) 29:327–39. doi: 10.1016/0277-9536(89)90281-5
15. Keys HM, Kaiser BN, Kohrt BA, Khoury NM, Brewster ART. Idioms of distress, ethnopsychology, and the clinical encounter in Haiti’s Central Plateau. Soc Sci Med. (2012) 75:555–64. doi: 10.1016/j.socscimed.2012.03.040
16. Llenas A. The color monster: A story about emotions. New York: Little, Brown and Company (2021).
17. Reynosa: El especialista de las emociones que habla con menores migrantes (2022).
Disponible en: https://www.youtube.com/watch?v=n0AvRy0AKrQ (Fecha de acceso: 13 de Octubre, 2025).
